# Anti-atherosclerotic function of Astragali Radix extract: downregulation of adhesion molecules *in vitro* and *in vivo*

**DOI:** 10.1186/1472-6882-12-54

**Published:** 2012-04-26

**Authors:** Yang You, Yan Duan, Shao-wei Liu, Xiao-lin Zhang, Xiu-li Zhang, Jia-tao Feng, Cheng-hui Yan, Ya-ling Han

**Affiliations:** 1 Department of Cardiology, Cardiovascular Research Institute, Shenyang Northern Hospital, Shenyang, China; 2 The Affiliated Hospital of Liaoning University of TCM, Shenyang, China; 3 Dalian Institute of Chemical Physics, Chinese Academy of Sciences, Dalian, China; 4 Department of Cardiology, Shenyang General Hospital, 83 Wenhua Road, Shenyang 110840, China

**Keywords:** Astragali Radix extract, Vascular cell adhesion molecule-1, Vntercellular adhesion molecule-1, Apolipoprotein E-deficient mice, Atherosclerosis

## Abstract

**Background:**

Atherosclerosis is considered to be a chronic inflammatory disease. Astragali Radix extract (ARE) is one of the major active ingredients extracted from the root of *Astragalus membranaceus* Bge. Although ARE has an anti-inflammatory function, its anti-atherosclerotic effects and mechanisms have not yet been elucidated.

**Methods:**

Murine endothelial SVEC4-10 cells were pretreated with different doses of ARE at different times prior to induction with tumor necrosis factor (TNF)-α. Cell adhesion assays were performed using THP-1 cells and assessed by enzyme-linked immunosorbent assay, western blotting and immunofluorescence analyses to detect the expression of vascular cell adhesion molecule-1 (VCAM-1), intercellular adhesion molecule-1 (ICAM-1), phosphorylated inhibitor of κB (p-iκB) and nuclear factor (NF)-κB. We also examined the effect of ARE on atherosclerosis in the aortic endothelium of apolipoprotein E-deficient (apoE^−/−^) mice.

**Results:**

TNF-α strongly increased the expression of VCAM-1 and ICAM-1 accompanied by increased expression of p-iκB and NF-κB proteins. However, the expression levels of VCAM-1 and ICAM-1 were reduced by ARE in dose- and time-dependent manners, with the strongest effect at a dose of 120 μg/ml incubated for 4 h. This was accompanied by significantly decreased expression of p-iκB and inhibited activation of NF-κB. Immunofluorescence analysis also revealed that oral administration of ARE resulted in downregulation of adhesion molecules and decreased expression of macrophages in the aortic endothelium of apoE^−/−^ mice. ARE could suppress the inflammatory reaction and inhibit the progression of atherosclerotic lesions in apoE^−/−^ mice.

**Conclusion:**

This study demonstrated that ARE might be an effective anti-inflammatory agent for the treatment of atherosclerosis, possibly acting via the decreased expression of adhesion molecules.

## Background

Atherosclerosis is one of the most prevalent diseases in the world and is characterized by multifactorial and multistep processes. Atherosclerosis is known to be a chronic inflammatory disease, although its pathophysiological mechanisms remain elusive [1]. The adhesion of circulating leukocytes to the endothelium is an important cellular response to inflammation. Endothelial cells express an array of adhesion molecules that mediate the process of leukocyte attachment to the vascular wall during inflammation [2]. Leukocyte-endothelium interactions require regulation of the expression of various adhesion molecules by endothelial cells, such as vascular cell adhesion molecule-1 (VCAM-1) and intercellular adhesion molecule-1 (ICAM-1) [3]. Proinflammatory mediators such as interleukin-1 and tumor necrosis factor (TNF)-α can promote the activation of endothelial cells in the early stages of the inflammatory process. Activation on endothelial cells results in increased expression of VCAM-1 and ICAM-1 on their surfaces, causing leukocytes to adhere to the endothelium at local sites of inflammation [4], and ultimately resulting in the development of atherosclerosis. Various inflammatory pathways have been implicated in atherosclerosis, leading not only to the recruitment and entry of inflammatory cells into the arterial wall, but also to modification of the morphology and composition of atherosclerotic plaques. Certain inflammatory pathways, such as those involving VCAM-1, and ICAM-1, appear to play an important role in lesion initiation [5]‐[7].

The root of *Astragalus membranaceus* Bge is a common Chinese medicinal herb. Several compounds isolated from this plant, as well as the plant itself, have been reported to show therapeutic potential in some diseases, such as nephropathy, cardiac contractile dysfunction and open wounds [8]‐[10], and the root of *A. membranaceus* Bge was been widely used for the treatment of cardiovascular diseases in ancient China. Astragali Radix extract (ARE) is one of the major active ingredients extracted from the root of *A. membranaceus* Bge. Previous studies showed that ARE had potential anti-inflammatory activity [9,11]‐[14], and recent evidence demonstrated that ARE could reduce macrophage migration [15] and adhesion to endothelial cells, and relieve local inflammation via a nuclear factor (NF)-κB pathway [16]‐[19]. However, the anti-atherosclerotic effects of ARE and its mechanisms have not yet been elucidated.

The present study therefore aimed to clarify the anti-atherosclerotic effects and molecular mechanisms of ARE by investigating its effects on the expression of adhesion molecules in murine endothelial cells stimulated with TNF-α, investigating its influences on the NF-κB pathway, and assessing the progression of atherosclerotic lesions and the expression of adhesion molecules in the aortic endothelium of ARE-treated apolipoprotein E-deficient (apoE^−/−^) mice.

## Materials and methods

### Reagents

ARE was provided by the Dalian Institute of Chemical Physics, Chinese Academy of Sciences (Dalian, China), and dissolved and diluted in incubation medium to yield final concentrations of 30‐120 μg/ml. TNF-α was purchased from Sigma-Aldrich (Saint Louis, MO, USA). Goat monoclonal anti-VCAM-1, goat monoclonal anti-ICAM-1, mouse monoclonal NF-κB p65, mouse monoclonal phosphorylated inhibitor of κB (p-iκB)-α, goat polyclonal Lamin B and anti-β-actin antibodies were purchased from Santa Cruz (CA, USA). Rat anti-mouse Mac3 and rat anti-mouse CD31 antibodies were purchased from BD Pharmingen (San Diego, CA, USA). The mouse soluble VCAM-1 immunoassay and mouse soluble ICAM-1 immunoassay kits were purchased from R&D Systems (MN, USA). The enhanced chemiluminescence (ECL) western blotting system was from Amersham Biosciences (Buckinghamshire, UK).

### Endothelial cell culture and treatment

The murine endothelial cell line (SVEC4-10) was purchased from the American Type Culture Collection (MD, USA) and cultured in DMEM supplemented with 10 % fetal calf serum (FCS, HyClone, UT, USA), 30 μg/ml endothelial cell growth factor (Sigma-Aldrich, Saint Louis, MO, USA), 5 U/ml heparin, 100 U/ml penicillin, and 100 U/ml streptomycin at 37°C in an atmosphere of 5 % CO_2_ and 95 % air. The phenotype of SVEC cells was confirmed morphologically and by positive immunofluorescence staining of CD31. Cells were treated with 30, 60, or 120 μg/ml ARE in fresh serum-free medium and incubated for 2, 4, 6, or 8 h.

### Culture of THP-1 cells

THP-1 cells were purchased from the American Type Culture Collection and grown in RPMI-1640 media (Sigma) containing 10 % FCS, 100 μg/ml streptomycin, 100 IU/ml penicillin, 250 ng/ml fungizone, 1 mM glutamine, 5 × 10^–5^ M 2-mercaptoethanol, and routinely subcultured three times per week at a ratio of 1:5.

### Mice

Male apoE^−/−^ mice (n = 24, age 8 weeks, 18–20 g) on a C57BL/6 J background were obtained from the Department of Laboratory Animal Science, Peking University Health Science Center, China. All mice were barrier housed, specific pathogen-free, and maintained in static microisolator cages. Mice were fed a high-fat, high-cholesterol diet containing 15 % fat and 0.25 % cholesterol. ApoE^−/−^ mice were randomized into either a control group or an ARE group (n = 12 for each group). ARE mice were given oral doses of ARE (5 g/kg body weight per day). Blood and tissues were collected at 24 weeks of age for further analysis. The care and use of animals were performed in adherence with the National Institutes of Health Guidelines, and all experimental procedures involving animals were approved by the Ethics Committee of Shenyang Northern Hospital.

### Adhesion assay for THP-1 cells

SVEC cells were plated in six-well plates and allowed to grow to confluence, as described above. The medium was removed and the cells were then incubated with 1.0 ml/well DMEM medium containing 10 % FCS with or without TNF-α (10 ng/ml) or TNF-α plus ARE at the concentrations and incubation times indicated above. Culture supernatant was removed from the treated cells at the indicated time points, and the cells were gently washed three times with DMEM medium. A volume of 1.0 ml of RPMI-1640 media containing 1 × 10^6^ THP-1 cells was then added to the endothelial cell monolayers in each well. The binding phase of the assay was performed at 37°C in a 5 % CO_2_ atmosphere for 15 min for THP-1 cells. Thereafter the wells were washed three times with phosphate-buffered saline (PBS) (1.0 ml/well), and all wells were examined under a microscope to determine if any loss of endothelial cells had occurred during incubation or washing. The number of THP-1 cells washed in PBS was counted using a counting slide. Experiments were performed in triplicate.

### Enzyme-linked immunosorbent assay (ELISA)

Aliquots of culture medium were collected from cells treated as described above. VCAM-1 and ICAM-1 levels in the culture medium were measured directly using an ELISA kit (R&D Systems), according to the manufacturer’s instructions. Standards containing known amounts of recombinant VCAM-1 and ICAM-1 were also analyzed. ELISA assay results were measured using a microplate reader. Absorbance was measured spectrophotometrically at 450 nm and plotted against a standard curve with VCAM-1 and ICAM-1 levels expressed in ng/ml. Experiments were performed in triplicate.

### Western blot analysis

Confluent cells were cultured in medium with or without TNF-α (10 ng/ml) or TNF-α plus ARE at the concentrations and the incubation times indicated above to detect VCAM-1, ICAM-1, NF-κB, and p-iκB. Total proteins were extracted with 100 μl of lysis buffer (50 mmol/l Tris, 10 mmol/l MgCl_2_, 0.5 mol/l NaCl, 1 % Triton X-100) supplemented with a protease inhibitor mixture and 1 mmol/l phenylmethylsulfonyl fluoride. Cells were maintained on ice and lysates were harvested by scraping. The supernatants were collected after centrifugation at 14,000 *g* for 10 min. For nuclear protein analysis, cells were resuspended in hypotonic buffer on ice and detergent was added to 0.5 %. Nuclei were pelleted and resuspended in nuclear protein extract buffer (50 mmol/l HEPES [pH 7.8], 300 mmol/l NaCl, 50 mmol/l KCl, 0.1 mmol/l EDTA, 10 % [v/v] glycerol) on ice for 30 min and then spun, and the supernatant was saved. Protein levels in the supernatant were determined using the BCA method. Aliquots of 30 μg of each protein sample were resolved by 10 % sodium dodecyl sulfate polyacrylamide-gel electrophoresis and transferred to a polyvinylidene fluoride membrane. After transfer, membranes were blocked with 5 % nonfat dry milk in Tris-buffered saline and then incubated overnight with primary antibodies at 4°C. After washing, blots were further incubated with 1:2,000 diluted secondary antibodies including horseradish peroxidase (HRP)-conjugated goat anti-mouse, goat anti-rabbit, or rabbit anti-goat IgG for 2 h at room temperature. After additional washing, HRP activity was detected by ECL, according to the manufacturer’s instructions.

### Quantitation of arterial atherosclerotic lesions

The proximal aortas of apoE^−/−^ mice were immersed in ice-cold 4 % paraformaldehyde for 30 min. The samples were then transferred to 30 % sucrose-PBS solution at 4°C for 24 h, embedded in tissue freezing medium and snap-frozen in liquid nitrogen. Cryosections (5 μm thick) were cut from the proximal aortas beginning at the end of the aortic sinus, and stained with oil red O and hematoxylin and eosin. Arterial atherosclerotic lesions were also measured in aortas by en face oil red O staining. Quantitative analysis of the lesions was performed using Image Pro Plus software on at least 15 sections from each animal by an operator who was blinded to group assignment.

### Immunofluorescence staining

SVEC cells were plated on coverslips and incubated at 37°C and 5 % CO_2_ to allow the cells to adhere and spread. Serial 5-μm-thick cryosections were cut from the proximal aortas. Cells or cryosections were fixed with 4 % paraformaldehyde for 20 min. After washing three times with PBS, cells or cryosections were permeabilized with 1 % Triton X-100 in PBS for 30 min and blocked with goat serum for 20 min at room temperature. The cells were stained with primary antibodies against NF-κB. The cryosections were incubated with primary antibodies to VCAM-1, ICAM-1 or Mac-3. Alexa Fluor 488, 555, and 568 secondary antibodies (Invitrogen, OR, USA) were used at concentrations of 1:300. Nuclei were stained with 4',6-diamidino-2-phenylindole (DAPI). Stained cells or tissues were observed using the Leica QWN (Wetzlar, Germany) analysis system.

### Assessment of cell viability

The 3-(4,5-dimethylthiazol-2-yl)-2,5-diphenyltetrazolium bromide (MTT; Sigma-Aldrich, Saint Louis, MO, USA) assay is based on the cleavage of MTT by mitochondrial dehydrogenases, reflecting the cell viability. SVEC cells were treated with 10 ng/ml TNF-α in the absence or presence of ARE (30, 60 or 120 μg/ml) for 8 h, followed by the addition of 0.5 mg/ml MTT medium and incubation for 4 h at 37°C. The medium was removed and dimethyl sulfoxide was added. The absorbance was examined at 570 nm.

### Statistical analysis

The data are expressed as mean ± SD. All data were analyzed using SPSS 13.0 statistical software. Differences between two groups were compared using unpaired Student’s *t*-tests. Differences among three or more groups were compared using one-way analysis of variance (ANOVA). Statistical significance was defined as P < 0.05 (two-tailed).

## Results

### ARE inhibits TNF-α-stimulated adhesion of THP-1 cells to SVEC cells

**Figure 1:**
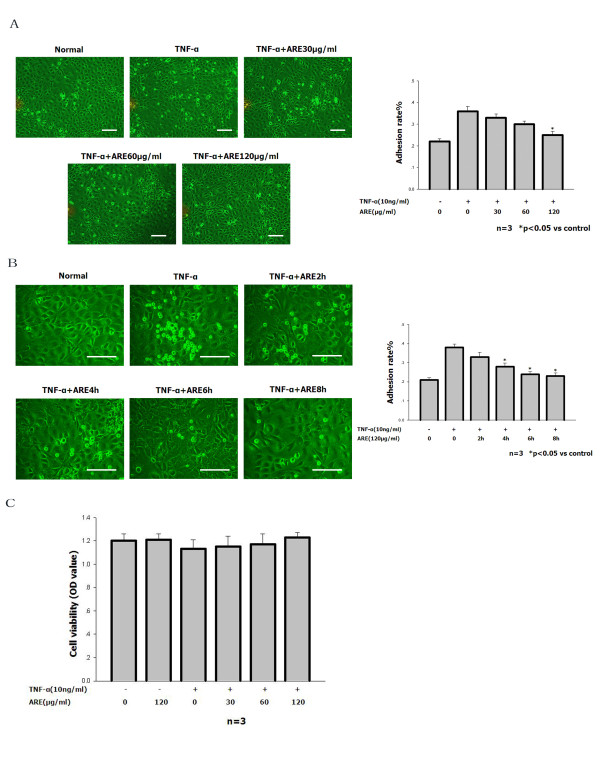
**ARE inhibited TNF-α-stimulated adhesion of THP-1 cells to SVEC cells. A, B.** Quantitation of THP-1 cells adhering to untreated SVEC cells and to SVEC cells treated for 4 h with 10 ng/ml TNF-α in the absence or presence of ARE (30, 60 or 120 μg/ml) for up to 2, 4, 6, or 8 h. For details of the adhesion assay see Materials and methods. The number of adherent THP-1 cells was determined using a counting slide. Data are representative of at least three independent experiments and are shown as mean values ± S.D. *P < 0.05 compared with cells treated with TNF alone. Scale bars: 50 μm. **C. **SVEC cells were treated with 10 ng/ml TNF-α in the absence or presence of ARE (30, 60 or 120 μg/ml) for 8 h. The viability of SVEC cells was determined by MTT analysis.

The number of THP-1 cells adhering to the endothelial cell monolayer was increased by pretreatment with 10 ng/ml TNF-α for 4 h. However, the TNF-α-stimulated increase in leukocyte adhesion was reduced almost to control levels when SVEC cells were treated with 10 ng/ml TNF-α together with 120 μg/ml ARE, especially after 4–8 h (Figure 1A and B). The MTT assay was used to exclude the possibility of toxic effects induced by ARE (Figure 1C). ARE had no influence on cell viability at the concentrations used. These result demonstrate that the downregulation of adhesion molecules by ARE in SVEC cells was not a result of direct toxicity of the substance.

### ARE inhibits upregulation of VCAM-1 and ICAM-1 expression induced by TNF-α

**Figure 2:**
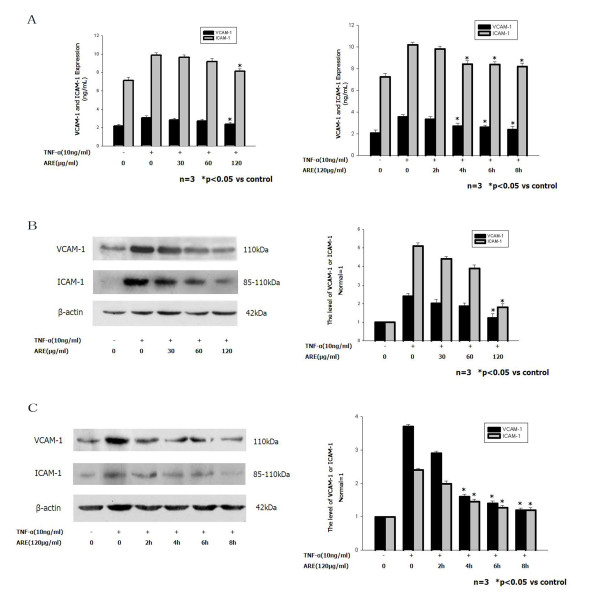
**ARE inhibited upregulation of VCAM-1 and ICAM-1 expression induced by TNF-α (ELISA and western blot).** Confluent SVEC cells were incubated with medium or 10 ng/ml TNF-α alone, or with 10 ng/ml TNF-α together with 30, 60 or 120 μg/ml ARE for 4 h. Confluent SVEC cells were preincubated with 120 μg/ml ARE for 2, 4, 6, or 8 h, and thereafter washed and incubated for 4 h with 10 ng/ml TNF-α. Culture medium and proteins were collected and analyzed for VCAM-1 and ICAM-1 expression by ELISA and western blot at the time points indicated. **A.** ELISA analyses of VCAM-1 and ICAM-1 expression levels in SVEC cells. **B.** Western blot analyses of VCAM-1and ICAM-1 expression levels in SVEC cells. VCAM-1 and ICAM-1 levels were normalized to the levels of β-actin.

ELISA assays demonstrated that the expression levels of soluble VCAM-1 and soluble ICAM-1 in the supernatant from SVEC cells increased rapidly after exposure to TNF-α (10 ng/ml) (Figure 2A). Expression levels of VCAM-1 and ICAM-1 induced by 10 ng/ml TNF-α were inhibited by co-incubation with different concentrations of ARE (30, 60 or 120 μg/ml) in a dose-dependent manner. The TNF-α-induced VCAM-1 and ICAM-1 expression levels were inhibited by 61 % and 67 %, respectively, by 120 μg/ml ARE. Preincubation of SVEC cells with 120 μg/ml ARE for 2, 4, 6, or 8 h resulted in inhibition of the TNF-α-induced VCAM-1 and ICAM-1 expression levels in a time-dependent manner. Significant reductions in TNF-α-induced VCAM-1 and ICAM-1 expression levels were seen in cells preincubated with ARE for 4–8 h. These results demonstrate that ARE inhibited the induction of VCAM-1 and ICAM-1 by TNF-α in dose- and time-dependent manners.

Western blot analysis showed similar results regarding the levels of VCAM-1 and ICAM-1 protein expression. ARE dose-dependently inhibited the TNF-α-induced increases in VCAM-1 and ICAM-1 protein levels; ARE 120 μg/ml completely abolished TNF-α-induced upregulation of VCAM-1 and ICAM-1 (Figure 2B). Preincubation of SVECs with 120 μg/ml ARE for 2, 4, 6, or 8 h inhibited TNF-α-induced VCAM-1 and ICAM-1 expression levels in a time-dependent manner. Significant reductions in TNF-α-induced VCAM-1 and ICAM-1 expression levels were seen in cells preincubated with ARE for 4–8 h (Figure 2C).

### ARE inhibits TNF-α-induced NF-κB activation

**Figure 3:**
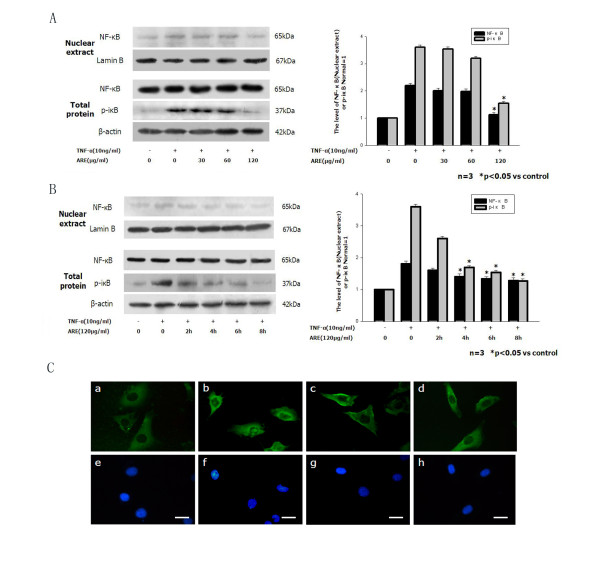
**ARE inhibited TNF-α induced upregulation of NF-κB expression (western blot) and translocation (immunofluorescence staining). A**. Confluent SVEC cells were incubated with medium or 10 ng/ml TNF-α alone, or with 10 ng/ml TNF-α together with 30, 60 or 120 μg/ml ARE for 4 h. **B**. Confluent SVEC cells were preincubated with 120 μg/ml ARE for 2, 4, 6, or 8 h, and thereafter washed and incubated for 4 h with 10 ng/ml TNF-α. NF-κB levels were normalized to the levels of β-actin. **C**. ARE inhibited TNF-α-induced NF-κB translocation from the cytoplasm to the nucleus in SVEC cells. SVEC cells were cultured on glass coverslips in six-well plates for 2 days, and then incubated with medium (control), 120 μg/ml ARE, or TNF-α (10 ng/ml) alone, or TNF-α in combination with ARE for 4 h. After stimulation, the cells were washed, fixed and permeabilized. The cells were then blocked with 2 % bovine serum albumin in PBS solution, and incubated with a mouse monoclonal NF-κB p65 antibody, followed by a second fluorescein isothiocyanate-conjugated antibody. Nuclei were stained with DAPI, and stained cells were assessed by fluorescence microscopy. Immunofluorescent images shown in **a–h** are representative of at least three experiments with determinations made in quadruplicate. **a**, untreated control cells; **b**, cells treated with TNF-α (10 ng/ml) alone; **c**, cells treated with ARE (120 μg/ml) alone; **d**, cells treated with TNF-α plus ARE; **e–h**, nuclei. Scale bars: 10 μm.

To determine if ARE-induced inhibition of adhesion-molecule expression was mediated by blocking the p-iκB/NF-κB pathway, the total protein level of p-iκB and the nuclear protein level of NF-κB p65 were analyzed by western blotting. The levels of p-iκB and NF-κB protein increased dramatically following induction by TNF-α (10 ng/ml). Co-incubation of the cells with different concentrations of ARE at different times prevented iκB phosphorylation and NF-κB activation in dose- and time-dependent manners. ARE almost completely abolished TNF-α- induced iκB phosphorylation and NF-κB activation after preincubation for 4–8 h at 120 μg/ml (Figure 3A and B). Furthermore, immunofluorescence staining demonstrated that ARE could inhibit TNF-α-induced translocation of NF-κB to the nucleus. NF-κB was detected in the cytoplasm in untreated control cells or cells treated with ARE alone (Figure 3C). However, rapid translocation of NF-κB from the cytoplasm to the nucleus occurred when the cells were incubated for 4 h with TNF-α (10 ng/ml). This translocation was significantly inhibited by 120 μg/ml ARE, and the nucleus remained virtually free of the transcription factor in cells treated with both TNF-α and ARE.

### ARE prevents atherosclerosis development in apoE^−/−^ mice

**Figure 4:**
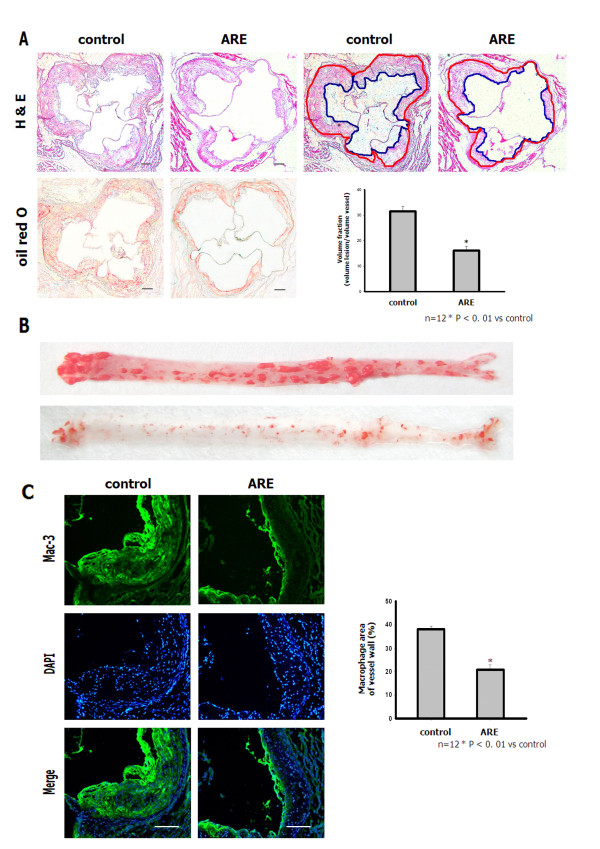
**ARE inhibited progression of established atherosclerotic lesions and accumulation of macrophages in the aorta of apoE**^**−/−**^**mice. A**. Hematoxylin and eosin and oil red O staining of lesions in the aortic root in control and ARE apoE^−/−^ mice (100×). Scale bars: 80 μm. **B**. Representative oil red O-stained aortas from apoE^−/−^ mice in both groups. Bar graph shows distribution of intima/lumen ratio. *P < 0.01 vs control. **C**. Atherosclerotic lesions in aortic root from control and ARE apoE^−/−^ mice immunostained with anti-mouse macrophage antibody (Mac-3). Double-immunofluorescence staining (n = 4–5 sections per tissue, at least three sites of analysis per slide). Bar graph shows quantitative comparison of composition of atherosclerotic lesions between control and ARE-treated groups. Scale bars: 80 μm.

All apoE^−/−^ mice fed a high-fat, high-cholesterol diet for 16 weeks developed atherosclerotic lesions along the proximal aortic walls and at the valve cusps. The intima was markedly thickened with the accumulation of lipids, macrophages and foam cells. However, ARE-treated mice showed a significant reduction in atherosclerotic-plaque-lesion size compared with control mice. The ratio of plaque area to total vessel area in the ARE mice was 16.12 ± 1.73 % compared with 31.51 ± 1.92 % in control mice (P < 0.01) (Figure 4A). In addition, en face oil red O-stained aortas from apoE^−/−^ mice in both groups exhibited similar results (Figure 4B). Atherosclerotic lesions in the aortic root in the control and ARE-treated apoE^−/−^ mice were immunostained with anti-mouse macrophage antibody (Mac-3). Macrophage infiltration into the endothelium was significantly inhibited in ARE-treated apoE^−/−^ mice (21.34 ± 1.82 %) compared with control apoE^−/−^ mice (38.4 ± 1.41 %) (Figure 4C).

### ARE decreases the expression of adhesion molecules in the endothelium of apoE^−/−^ mice

**Figure 5:**
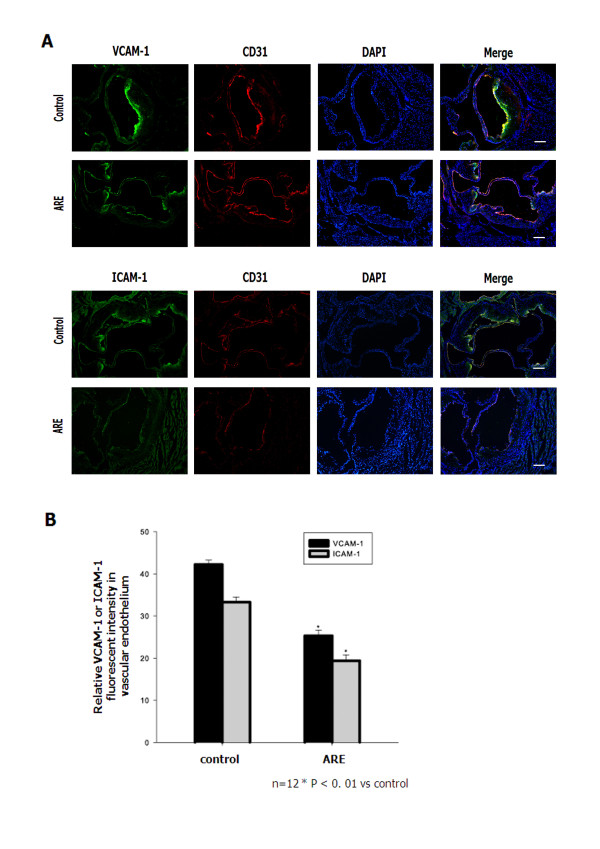
**ARE decreased the expression of adhesion molecules in the endothelium of apoE**^**−/−**^**mice. A**. ARE treatment suppressed the levels of VCAM-1 and ICAM-1 in the aortic endothelium of apoE^−/−^ mice. Double-immunofluorescence staining (n = 4–5 sections per tissue, at least three sites of analysis per slide). **B**. The relative fluorescence intensity of VCAM-1 and ICAM-1 in the endothelium of apoE ^−/−^ mice. *P < 0.01 vs control. Scale bars: 80 μm.

We investigated the effects of ARE on inflammatory responses to further clarify the molecular mechanisms whereby ARE restricts the development of atherosclerotic lesions. The levels of adhesion molecules in the endothelium of apoE^−/−^ mice were evaluated. Immunofluorescence analysis revealed reduced staining of VCAM-1 and ICAM-1 in aortic sections from ARE-treated apoE^−/−^ mice. The relative fluorescence intensities of VCAM-1 and ICAM-1 in the endothelium of ARE-treated apoE^−/−^ mice were significantly reduced (Figure 5). Staining for CD31 showed that the aortic endothelium remained intact in ARE-treated mice. These results suggest that ARE inhibited the interaction of inflammatory cells with endothelial cells.

## Discussion

Multiple lines of evidence support the idea that atherosclerosis is a chronic inflammatory disease [1]. In China, *A. membranaceus* Bge root has been widely used for the treatment of cardiovascular diseases [20,21], and ARE is one of the major active ingredients extracted from this root. Previous studies have revealed that ARE possesses anti-inflammatory and immunoregulatory roles [22]. Furthermore, recent evidence demonstrated that ARE could reduce macrophage migration [15] and adhesion to endothelial cells, and relieved local inflammation via the NF-κB pathway [16]‐[19]. We hypothesized that ARE might have anti-atherosclerotic properties associated with its anti-inflammatory role. We therefore investigated the effects of ARE on the expression of adhesion molecules and its anti-inflammatory effects *in vitro* and *in vivo*. ARE inhibited the activation of NF-κB in SVEC cells *in vitro* and reduced atherosclerotic-lesion size in apoE^−/−^ mice fed a high-fat, high-cholesterol diet *in vivo*.

One of the initial steps in the inflammatory cascade is the expression of adhesion molecules in endothelial cells and the adhesion of circulating leukocytes to the activated endothelium [23,24]. The current study found that pretreatment of SVEC cells with ARE almost abolished TNF-α-induced adhesion of monocytic THP-1 cells to endothelial cells. ARE could reduce TNF-α-induced upregulation of VCAM-1 and ICAM-1 expression levels in dose- and time-dependent manners, suggesting that ARE might be able to block leukocyte adhesion *in vitro* completely. Co-treatment of SVEC cells with TNF-α and ARE resulted in dose- and time-dependent decreases in adhesion molecules accompanied by a significant decrease in p-iκB expression and inhibition of NF-κB activation. NF-κB binds to specific sites in the promoter region of target genes and apparently mediates TNF-α-induced activation of endothelial cells by transiently inducing the genes coding for adhesion molecules [25,26] and atherosclerosis [27]‐[31]. ARE dose- and time-dependently inhibited TNF-α-induced upregulation of NF-κB and p-iκB expression, indicating that it interfered with the NF-κB pathway by inhibiting TNF-α-induced degradative phosphorylation of iκB, an inhibitor of NF-κB, which binds to the transcription factor in the cytosol, thereby preventing its translocation to the nucleus [32]. The current results showed that ARE at 120 μg/ml could completely inhibit TNF-α-induced NF-κB nuclear translocation. Selective inhibition of NF-κB activation by ARE would therefore affect VCAM-1 and ICAM-1 preferentially. We demonstrated that ARE regulated VCAM-1 and ICAM-1 expression levels through down-regulation of the NF-κB pathway in SVEC cells.

To further elucidate the impact of ARE on atherosclerosis, we investigated its effects in apoE^−/−^ mice fed on an atherogenic diet. ApoE^−/−^ mice are created by targeted gene inactivation, and are considered to be a relevant model for atherosclerosis because they are hypercholesterolemic and develop spontaneous arterial lesions [33]. ApoE^−/−^ mice treated with ARE had significantly smaller aortic sinus lesions, lower numbers of macrophages in the aortic wall, and decreased expression levels of adhesion molecules (VCAM-1 and ICAM-1) in the vessel wall compared with control mice. In conclusion, ARE could prevent the formation of atherosclerosis by regulating the expression of adhesion molecules in aortas of apoE^−/−^ mice.

## Conclusions

In conclusion, the results of this study demonstrate that ARE has the capacity to protect the aorta from atherosclerosis, potentially via an anti-inflammatory mechanism. To the best of our knowledge, this is the first study to reveal a possible role for ARE in the treatment of atherosclerosis. One of the major mechanisms of ARE action appears to be through regulating the levels of adhesion molecule expression in endothelial cells via the NF-κB pathway. These findings suggest an anti-atherosclerotic effect of ARE and support its potential clinical use for the treatment of atherosclerosis.

## Competing interests

The authors declare that they have no competing interests.

## Authors’ contributions

YY was responsible for performing the experiments, analyzing data and drafting the manuscript. YD and SL participated in the analysis of data. XZ^c^ and JF extracted Astragali Radix extract. CY, XZ^a^ and YH supervised the whole study and revised the manuscript. All authors have read and approved the final manuscript.

## Pre-publication history

The pre-publication history for this paper can be accessed here:

http://www.biomedcentral.com/1472-6882/12/54/prepub
